# Efficacy and safety of video double-lumen tube intubation in lateral position in patients undergoing thoracic surgery: a randomized controlled trial

**DOI:** 10.1186/s12871-024-02567-w

**Published:** 2024-05-20

**Authors:** Qianqian Rao, Hong Yu, Ping Li, Gongwei Zhang, Jun Zeng, Qiang Pu, Hai Yu

**Affiliations:** 1 Department of Anesthesiology, West China Hospital, Sichuan University, Chengdu, 610041 China; 2 Department of Anesthesiology, Wu’an First People’s Hospital, Handan, China; 3 Department of Anesthesiology, West China (Airport) Hospital, Sichuan University, Chengdu, China; 4 Department of Thoracic Surgery, West China Hospital, Sichuan University, Chengdu, China

**Keywords:** Video double-lumen tube, Intubation, Intratracheal, One-lung ventilation, Patient positioning, Thoracic surgery

## Abstract

**Background:**

Video double-lumen tube (VDLT) intubation in lateral position is a potential alternative to intubation in supine position in patients undergoing thoracic surgery. This non-inferiority trial assessed the efficacy and safety of VDLT intubation in lateral position.

**Methods:**

Patients (18–70 yr) undergoing right thoracoscopic lung surgery were randomized to either the left lateral position group (group L) or the supine position group (group S). The VDLT was placed under video larygoscopy. The primary endpoint was the intubation time. Secondary endpoints included VDLT displacement rate, intubation failure rate, the satisfaction of surgeon and nurse, and intubation-related adverse events.

**Results:**

The analysis covered 80 patients. The total intubation time was 52.0 [20.4]s in group L and 34.3 [13.2]s in group S, with a mean difference of 17.6 s [95% confidence interval (CI): 9.9 s to 25.3 s; *P* = 0.050], failing to demonstrate non-inferiority with a non-inferiority margin of 10 s. Group L, compared with group S, had significantly lower VDLT displacement rate (*P* = 0.017) and higher nurse satisfaction (*P* = 0.026). No intubation failure occurred in any group. Intubation complications (*P* = 0.802) and surgeon satisfaction (*P* = 0.415) were comparable between two groups.

**Conclusions:**

The lateral VDLT intubation took longer time than in the supine position, and non-inferiority was not achieved. The incidence of displacement as the secondary endpoint was lower in the L group, possibly due to changing body positions beforehand. The indication of lateral VDLT intubation should be based on a balance between the safety of airway management and the lower incidence of displacement.

**Trial registration:**

The study was registered at Chictr.org.cn with the number ChiCTR2200064831 on 19/10/2022.

**Supplementary Information:**

The online version contains supplementary material available at 10.1186/s12871-024-02567-w.

## Background

The double-lumen tube (DLT) is the most prevalent device employed for one-lung ventilation (OLV) during video-assisted thoracoscopic surgery (VATS) [1, 2], with the aim of ensuring a well-collapsed lung for optimal visualization of the surgical field [3–6]. The video double-lumen tube (VDLT), compared with conventional DLT, features an integrated imaging camera located at the end of the tracheal lumen, which enables continuous airway visualization, ensures the precision of tube placement during intubation and reduces the need for fiberoptic bronchoscopy, shortening the time required for intubation [1, 7–9]. However, malposition of either the traditional DLT or the VDLT may arise during shifting patient from supine to lateral position for surgery, impacting the efficiency of lung isolation [10, 11]. Recently, two clinical studies have proved that lateral DLT intubation reduced the incidence of DLT malposition for patients undergoing thoracic surgery compared to supine DLT intubation [12, 13]. As a result, VDLT intubation undertaken directly in the lateral position may be an alternative.

Prior researches have demonstrated the feasibility of single-lumen tube [14, 15] and laryngeal mask airway intubation [16, 17] in the lateral position, with a comparable intubation time and success rate between patients placed in the supine and lateral positions. However, larger size of DLT combined with deterioration of laryngoscopic view raise the difficulty of DLT intubation in the lateral position. It was evidenced by the recent two RCTs which found that the lateral DLT intubation was associated with longer intubation attempts and a more frequent failure rate [12, 13]. Therefore, the feasibility of lateral VDLT intubation in terms of intubation time for patients undergoing thoracic surgery still need further study.

We designed a prospective randomized noninferiority clinical trial to evaluate the efficacy and safety of VDLT intubation in lateral position in patients undergoing right thoracoscopic lung surgery. We tested the hypothesis that VDLT intubation in lateral position would be non-inferior to supine position in terms of intubation time. We further hypothesized that VDLT intubation in lateral position would decrease the incidence of VDLT displacement and not increase the incidence of intubation failure and intubation-related complications.

## Materials and methods

This single-center, prospective, randomized controlled, non-inferiority clinical trial was approved by the Institutional Review Board of Ethics Committee on Biomedical Research at West China Hospital of Sichuan University and registered at Chictr.org.cn (Trial number: ChiCTR2200064831; Date of registration: 19/10/2022). This study was performed in adhere to the applicable guideline: the consolidated standards of reporting trials (CONSORT). Written informed consent was obtained from all the participants before surgery.

### Patients

The inclusion criteria were as follows: aged 18–70 years old, American Society of Anesthesiologists (ASA) physical status 1 or 2, scheduled to undergo elective right thoracoscopic lung surgery and requiring a left lateral position. Patients with body mass index (BMI) > 30 kg/m^2^, anticipated difficult airway, limited neck motion, intraluminal lesions in the left main bronchus, an anatomical problem in the tracheobronchial tree, cardiopulmonary impairment, and patients at potential risk of reflux aspiration were excluded from the study.

### Randomization and blinding

The patients were allocated to either the left lateral position group (group L) or the supine position group (group S) in a 1:1 ratio through a random number table generated by a computer. The random allocation sequence was placed in a sequentially coded, sealed, and opaque envelope, which was opened by the anesthetist before anesthesia induction in the operating room. While the study was blinded to data collection personnel, it was not blinded to patients, surgeons, nurses and anesthetists responsible for treating patients.

### Anesthesia procedure and quality control

Standard monitoring protocols in thoracic surgery were followed. All patients received continuously monitoring, including saturation of pulse oxygen (SpO_2_), electrocardiogram (ECG), non-invasive or invasive blood pressure, capnography, neuromuscular and Bispectral Index (BIS) monitoring. Before induction of anesthesia, patients assigned to group S were positioned in a supine position, while those in group L were positioned laterally with right arm extended to facilitate mask ventilation and intubation (Supplementary file 1). All patients received preoxygenation for 10 min before induction. Anesthesia was induced intravenously with propofol 1.5–2.0 mg kg ^−1^, sufentanyl 0.2–0.3 µg kg ^−1^, and rocuronium 1 mg kg ^−1^. A skilled anesthetist with over 10 years experience in anesthesia for thoracic surgery inserted a left-sided VDLT (NORGAS, Jiangxi Norgas Medical Ltd., Jiangxi, China) through the vocal cords under the Macintosh laryngoscope.After removing the stylet, the VDLT was advanced and rotated 90° clockwise until its appropriate position was showed on the VDLT’s screen. Left side 32 or 35 Fr VDLTs were used in female patients and 35 or 37 Fr VDLTs in male patients. General anesthesia was maintained with either volatile (sevoflurane or desflurane) or propofol (targeting a BIS value between 40 and 60) and remifentanil with sufentanyl boluses as required. Lung protective ventilation was used in all patients’ ventilation management.

### The outcomes and definition

The primary outcome was the intubation time. Time to intubation was defined as the time from the insertion of larygoscopic blade into the mouth until the satisfactory placement in the endobronchial lumen achieved confirmed through the video of VDLT. Two time points were recorded, as follows: T1, glottis identification time, was defined as the time from the larygoscopic blade passing between the patient’s lips to identification of glottis; T2, railroading time, was defined as the time from identification of glottis to confirmation of bronchial tube positioning.

**Fig. 1:**
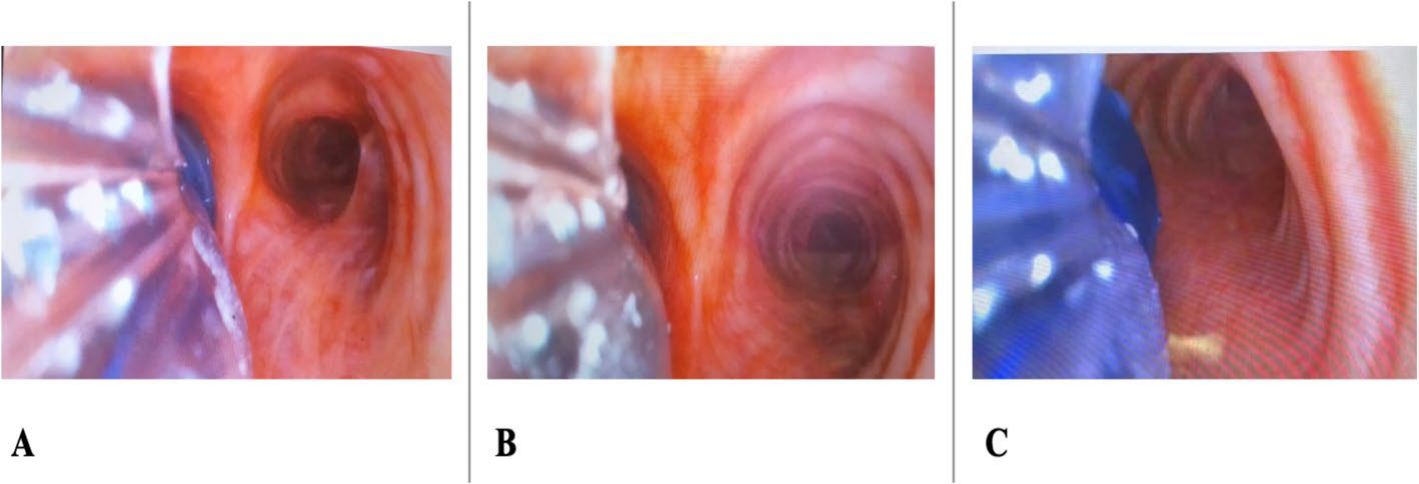
The different locations of the endotracheal tube are showed in the photograph. **A**. The appropriate location of the endotracheal tube. **B**. The bronchial cuff’s edge was totally hidden at the main bronchus entry’ and the cuff could not be seen on the screen. **C**. Bronchial cuff herniating into the carina and the carina could not be seen on the screen

The secondary outcomes included: (1) VDLT displacement rate: Fig. 1A depicts the endotracheal tube in its appropriate location. When bronchial cuff’s edge was completely concealed at the main bronchus’ entry and the cuff could not be seen on the camera screen (Fig. 1B) or when the cuff of the bronchus herniating into the carina and the carina could not be seen (Fig. 1C), the VDLT displacement was detected; (2) intubation failure rate: intubation was deemed failure when it could not be accomplished within 150 s or SpO_2_ dropped to 92% or below. The rescue method would be performed if a tracheal intubation failure happens. When the first intubation attempt failed, the anesthetist immediately performed mask ventilation. The patients would be changed from lateral to supine position if the second intubation attempt failed; (3) the satisfaction of surgeon and nurse: at the end of surgery, the surgeon and nurse were questioned the overall satisfaction of lung isolation and patient position. Satisfaction was ranked as follows: 0 = very dissatisfied, 1 = dissatisfied, 2 = somewhat satisfied, 3 = moderate satisfied, 4 = highly satisfied; (4) intubation-related adverse events: the events included mucosal trauma, lip or dental injuries, esophageal intubation, hoarseness and sore throat 30 min and 24 h after extubation. Sore throat was graded as mild (pain with swallowing), moderate (persistent pain and increasing with swallowing), and severe (pain interfering with eating and require analgesic medication).

### Sample size calculation

Sample size calculation was based on the hypothesis that intubation time in group L is not inferior to that in group S. Using a non-inferiority margin of 10 s, based on the findings of previous research [18, 19], the minimum sample size was calculated to be 80 patients to achieve a power of 80% and a 2.5% risk of a type I error (one-side test). Non-inferiority would have been declared if the upper limit of the 97.5% confidence interval (CI) of the mean difference (VDLT intubation time in group L minus that in group S) in the intubation time was below 10 s.

### Statistical analysis

All data were recorded in a Microsoft Excel database and all statistical analyses were performed using SPSS Statistics 26 (IBM SPSS Statistics for Windows, IBM Corp, Armonk, NY, USA). Kolmogorov–Smirnov test was used to test for normality of distribution. Continuous variables are presented as mean and standard deviation or median and interquartile range. Normally distributed continuous data comparisons were performed using independent t-test. Nonparametric analysis for non-normally distributed continuous variables was performed using the Mann–Whitney U test. Categorical data were shown as number and percentage and analyzed using Chi-square test or Fisher’s exact test. Non-inferiority was assessed for the primary outcome by one-sided 97.5%CI of absolute difference using the independent t-test. The results were considered significant with *P* < 0.05.

## Results

### Characteristics of the study population

**Fig. 2:**
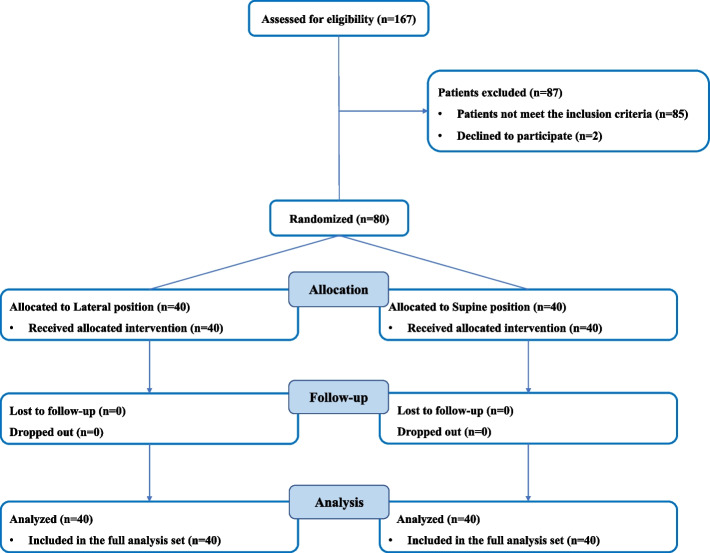
CONSORT flow diagram of the study

From January 2023 to June 2023, a total of 167 patients undergoing elective VATS were assessed for eligibility. 85 patients did not meet the inclusion criteria and 2 patients refused to participate. Consequently, a total of 80 patients who gave informed consent were enrolled in the study and randomly assigned to either group L (*n* = 40) or group S (*n* = 40). There were no patients lost to follow-up or dropped out, so we analyzed data from 80 patients (Fig. 2). Table 1 shows the patient characteristics and preoperative information. There were no statistically significant differences in baseline characteristics and preoperative data between two groups. The SpO_2_ before intubation in group L is lower than that in group S [97 (96–98) vs 98 (97–99), *p* = 0.043].

**Table 1:** Patient characteristics and preoperative data

	^Group L (*n*=40)^	^Group S (*n*=40)^
^Age, years^	^55 (51–62)^	^54 (50–60)^
^Sex (male), *n* (%)^	^13 (32.5)^	^9 (22.5)^
^Weight, kg^	^60.3±7.9^	^59.3±8.7^
^BMI, kg/m2^	^23.3±2.5^	^23.0±2.5^
^ASA physical status, I/II, *n* (%)^	^18 (45.0)/22 (55.0)^	^13 (32.5)/27 (67.5)^
^Smoking history, *n* (%)^	^5 (12.5)^	^6 (15.0)^
^Pulmonary function test^		
^FEV1, %^	^107.3±11.4^	^107.1±15.5^
^FVC, %^	^110.5±11.3^	^109.9±16.1^
^FEV1/FVC, %^	^80.9 (76.4–83.1)^	^79.6 (77.3–85.5)^
^SpO2 in room air, %^	^97 (96–98)^	^98 (97–99)^
^Interincisor Distance, cm^	^4.0 (4.0–4.5)^	^4.0 (4.0–5.0)^
^Thyromental Distance, cm^	^6.0 (6.0–8.0)^	^6.0 (6.0–9.0)^
^Sternomental Distance, cm^	^12.0 (12.0–15.0)^	^12.0 (12.0–14.0)^
^Mallampati score, I/II/III, *n* (%)^	^9 (22.5)/16(40.0)/15(37.5)^	^3 (7.5)/11(27.5)/25(65.0)^

Data are presented as the mean ± SD, median (interquartile range), or number (%)

*ASA* American Society of Anesthesiologists, *BMI* Body Mass Index, *FEV1* Forced Expiratory Volume in the first second, *FVC* Forced Vital Capacity, *NYHA* New York Heart Association, *SpO*_*2*_ saturation of pulse oxygen, *OLV* one-lung ventilation

Group L: endotracheal intubation with patients in the left lateral position; Group S: endotracheal intubation with patients in the supine position

### Primary outcome and intubation parameters

**Fig. 3:**
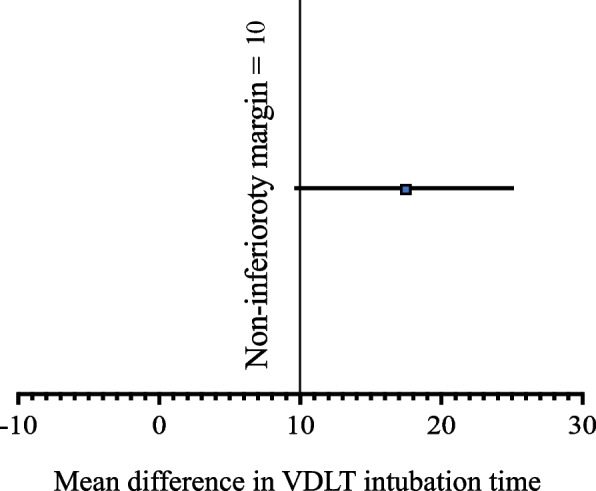
Mean difference in VDLT intubation time. The data are plotted as mean ± 95% CI. Vertical line at 10 represents margin of non-inferiority for VDLT Intubation time. VDLT: Video double-lumen tube; CI: confidence interval

The times to intubation were observed as 52.0 ± 20.4 s in the group L and 34.3 ± 13.2 s in the group S, respectively. The mean difference of the intubation time between two groups were 17.6 (95% CI 9.9 to 25.3), and the upper confidence boundary was greater than the non-inferiority margin of 10 s (non-inferiority *P* = 0.050) (Table 2 and Fig. 3). The glottis exposure time and railroading time in group L were longer than those in group S (11.1 ± 6.5 s vs 6.2 ± 2.6 s, *p* < 0.001; 40.9 ± 16.6 s vs 28.1 ± 11.9 s, *p* < 0.001, respectively). In group S, all cases were successfully intubated in the first attempt, while only 36 of 40 cases were successfully intubated in the first attempt in group L (*p* = 0.116). All the 4 cases failed attemptes at intubation in the group L were attributed to the inappropriate angulation of the VDLT. There was no statistically significant difference between the two groups in terms of the lowest SpO_2_ during VDLT intubation (Table 2).

**Table 2:** Intubating parameters

	Group L (*n* = 40)	Group S (*n* = 40)	Mean difference (95%CI)	*P *value
Total intubation time, s	52.0 ± 20.4	34.3 ± 13.2	17.6 (9.9 to 25.3)	0.050*
Glottis identification time, s	11.1 ± 6.5	6.2 ± 2.6	4.9 (2.7 to 7.1)	< 0.001
Railroading time, s	40.9 ± 16.6	28.1 ± 11.9	12.7 (6.3 to 19.2)	< 0.001
The lowest SpO_2_ during intubation, %	100 (99–100)	100 (99–100)	-	0.790
Number of attempts (1/2/3), *n* (%)	36(90)/4(10)/0(0)	40(100)/0(0)/0(0)	-	0.116

Data are presented as the mean ± SD, median (interquartile range], or number of patients (%)

*VDLT* Video Double-lumen tube, *OLV* One-lung ventilation

^*^Test results for the non-inferiority hypothesis

Group L: Endotracheal intubation with patients in the left lateral position; Group S: Endotracheal intubation with patients in the supine position

### Secondary outcomes

The rate of VDLT displacement was significantly lower in the group L than that in the group S (20% vs 47.5%, *p* = 0.017). There was no failed intubation case in either group S or L. No significant difference was observed between the two groups regarding surgeon satisfaction (*p* = 0.415) (Table 3). Group L received higher nurse satisfaction (*p* = 0.026) (Table 3). Considering the intubation-related adverse events, no statistical difference was observed in the incidence of hoarseness and sore throat 30 min and 24 h after extubation between two groups (Table 3). In addition, no incidence of esophageal intubation occurred in both groups. There was no significant difference in the occurrence of lips or dental injury and mucosal trauma (*p* = 0.802) (Table 3).

**Table 3:** Satisfaction evaluation and adverses effects

	Group L (*n* = 40)	Group S (*n* = 40)	*P* value
VDLT displacement, *n* (%)	8 (20.0)	17 (47.5)	0.017
Intubation failure, *n* (%)	0 (0.0)	0 (0.0)	1.000
Surgeon satisfaction^a^ (0/1/2/3/4)	0/0/5/10/25	0/1/2/17/20	0.415
Operation nurse satisfaction^a^ (0/1/2/3/4)	0/0/2/10/28	0/0/4/18/18	0.026
Hoarseness 30 min after extubation, *n* (%)			0.588
None	21 (52.5)	17 (42.5)	
Noticed by the patient	4 (10.0)	7 (17.5)	
Noticed by the observer	14 (35.0)	16 (40.0)	
Aphonia	1 (2.5)	0 (0.0)	
Sore throat 30 min after extubation^b^, *n* (%)			0.703
None	30 (75.0)	30 (75.0)	
Mild pain^c^	8 (20.0)	2 (5.0)	
Moderate pain	2 (5.0)	8 (20.0)	
Severe pain	0 (0.0)	0 (0.0)	
Hoarseness 24 h after extubation, *n* (%)			0.357
None	24 (60.0)	29 (72.5)	
Noticed by the patient	8 (20.0)	3 (7.5)	
Noticed by the observer	8 (20.0)	8 (20.0)	
Aphonia	0 (0.0)	0 (0.0)	
Sore throat 24 h after extubation^b^, *n* (%)			0.383
None	34 (85.0)	31 (77.5)	
Mild pain	5 (12.5)	7 (17.5)	
Moderate pain	1 (2.5)	2 (5.0)	
Severe pain	0 (0.0)	0 (0.0)	
The other intubation-related adverse events^d^, *n* (%)			0.802
Mucosal trauma	3 (7.5)	1 (2.5)	
Lips or dental injury	1 (2.5)	2 (5.0)	
Esophageal intubation	0 (0.0)	0 (0.0)	

Data are presented as number of patients (%)

^a^Satisfaction: 0 = very dissatisfied, 1 = dissatisfied, 2 = somewhat satisfied, 3 = moderate satisfied, 4 = highly satisfied

^b^Sore throat was graded as mild (pain with swallowing), moderate (persistent pain and increasing with swallowing), and severe (pain interfering with eating and require analgesic medication)

^c^The difference between the two groups was statistically significant, *p* < 0.05

^d^The other intubation-related adverse events include mucosal trauma, lip or dental injuries, esophageal intubation, laryngeal spasm, bronchospasm and arrhythmia

Group L: Endotracheal intubation with patients in the left lateral position; Group S: Endotracheal intubation with patients in the supine position

## Discussion

In this study, a non-inferiority test was conducted to compare VDLT intubation in patients undergoing right thoracoscopic lung surgery placed in lateral and supine pisitions. Our study results showed that the intubation time performed by skilled anesthetists in lateral position was longer than that in supine position with a mean difference of 17.6 s, the non-inferiority was not established (a non-inferior margin of 10 s) [18, 19]. However, the rate of VDLT displacement was significantly lower in patients placed in lateral position than that in supine position. Other findings including the lowest SpO_2_ during VDLT intubation, surgeon satisfaction and intubation complications were not different between two groups, while group L received higher nurse satisfaction.

The results were comparable to tracheal intubation time after airway scope in the lateral position [15], light wand-assisted intubation in the lateral position [14], and laryngeal mask airway facilitating tracheal intubation in the lateral position [14, 16]. However, in our study, we discovered that the mean VDLT intubation time in group L was longer than that in group S. These findings are consistent with pre-existing research, which suggests that tracheal intubation in the lateral position is more time-consuming and challenging than in supine position [20, 21]. Deterioration of laryngoscopic view and larger size of DLT raise the difficulty of intubation in the lateral position [22, 23]. In this trial, unsuccessful intubation occurred in four patients in the first attempt in group L, and a second attempt was success after reshaping the distal curve of VDLT. The proper angulation and shape of VDLT may facilitate VDLT intubation in the lateral position. It has been discovered that angling the bronchial lumen to a hockey stick shape was identified time-saving when intubating a left-sided DLT [24]. Our results showed that glottis exposure through video laryngoscopy in lateral position seems to be more difficult and time-consuming than when patients are in supine position. These results are comparable to those reported by Nileshwar et al. [25] McCaul et al. also revealed that the left lateral position has a detrimental effect on laryngoscopic view in 35% of patients [26]. BURP (backward, upward, rightward pressure) manoeuvre is broadly considered as an aid to bring vocal cords into view and improve vision during laryngoscopy [25]. Even if non-inferiority was not established, the average intubation time in group L was less than one minute. Additionally, no patients in lateral position experienced hypoxia, and the lowest SpO_2_ during intubation was comparable between the two groups. Therefore, with adequate preoxygenation, VDLT intubation in the lateral position may be feasible to a certain extent.

In our investigation, VDLT intubation in lateral position showed a significantly lower rate of VDLT displacement. It could be explained by the fact that intubation in lateral position eliminated the need to shift position from supine to lateral and mitigated neck movement, which in turn decreased the incidence of VDLT displacement. Tae-Gyoon Yoon et al. stated that the displacement of DLT may be closely associated to neck movement during the transition from supine to lateral position [27]. Notably, nurse satisfaction in group L was markedly higher than in group S. This might be linked to the patient’s ability to cooperate with the lateral position when awake, which is more labor-saving. Surgeon satisfaction was comparable between two groups probably because surgeons were more concerned about lung isolation’s effectiveness. Moreover, in our study, intubaton in the lateral position did not increase the incidence of intubation-related adverse events. The results were consistent with previous two studies which revealed a comparable incidence of airway complications, such as sore throat, hoarseness, oral mucosal trauma, and dental injury in each position in patient intubated with single-lumen tube [15, 17].

The study has some limitations that should be mentioned. First, the predetermined margin of non-inferiority in our study might be too small. The non-inferiority was not established when the margin was set as 10 s, which may not be clinically meaningful. Because no intubation failure occurred and the lowest SpO_2_ during intubation was comparable between two groups. Second, these excellent results in lateral position depend on the great experience of the anesthesiologists who participated in this one and therefore it is not extrapolated from all anesthesiologists. It might take longer than we reported, especially for less-experienced clinicians, as most anesthetic practitioners conventionally perform VDLT intubation in supine position and not routinely in lateral position. Third, only 80 patients from a single center were enrolled in our investigation and larger clinical trials including more patients from multiple centers are required to confirm the findings reported here. Forth, our restriction of the inclusion criteria to only right-side VATS and to patients with no airway difficulties can potentially limit the generalizability of the present results. This technique in lateral position does not seem optimal for obese patients or those with difficult airway, so it would have numerous contraindications. It is crucial to further confirm whether VDLT intubation in the lateral position is feasible for other patients population.

## Conclusions

The lateral VDLT intubation took longer time than in the supine position, and non-inferiority was not achieved. The incidence of displacement as the secondary endpoint was lower in the L group, possibly due to changing body positions beforehand. The indication of lateral VDLT intubation should be based on a balance between the safety of airway management and the lower incidence of displacement.

### Supplementary Information

Additional file 1: Supplementary file 1. VDLT intubation in the left lateral position

## Data Availability

The datasets used and/or analyzed during the current study are available from the corresponding author on reasonable request.

## Abbreviations

ASA: American Society of Anesthesiologists
BMI: Body mass index
BIS: Bispectral Index
CI: Confidence interval
CONSORT: Consolidated standards of reporting trials
DLT: Double-lumen tube
ECG: Electrocardiogram
OLV: One-lung ventilation
VDLT: Video double-lumen tube
VATS: Video-assisted thoracoscopic surgery
SpO_2_: Saturation of pulse oxygen
