# Supplementary material for: Efficacy and safety of video double-lumen tube intubation in lateral position in patients undergoing thoracic surgery: a randomized controlled trial
Source: BMC Anesthesiol. 2024 May 20;24:179. doi: 10.1186/s12871-024-02567-w (PMC11104002; doi:10.1186/s12871-024-02567-w)
Supplement: Supplementary file 1 — Additional file 1: Supplementary file 1. VDLT intubation in the left lateral position [file 12871_2024_2567_MOESM1_ESM.docx]

VDLT intubation with patient in the left lateral position

1. Mask ventilation with patient in the left lateral position with abduction of right arm. B. VDLT intubation completed with patient in the left lateral position with abduction of right arm.
